# Transcutaneous Neuromodulation for Constipation and Fecal Incontinence in Children: A Systematic Review and Meta-Analysis

**DOI:** 10.3390/life13020430

**Published:** 2023-02-02

**Authors:** Ronny Rodrigues Correia, Luis Felipe Orsi Gameiro, Nathalia Grion Trevisane, Matheus Bertanha, Erika Veruska Paiva Ortolan, Pedro Luiz Toledo de Arruda Lourenção

**Affiliations:** 1 Botucatu Medical School, São Paulo State University—Unesp, Araraquara 01049-010, Brazil; 2 Unimetrocamp University Center, Campinas 13035-500, Brazil

**Keywords:** constipation, fecal incontinence, neuromodulation, children

## Abstract

Introduction: Constipation is a disorder with a multifactorial origin. Constipation has a varied clinical presentation, including infrequent defecation of bulky stools and episodes of retentive fecal incontinence. Neuromodulation has been used to treat many health problems, with promising results. Objective: To conduct a systematic review of randomized clinical trials based on the effects of transcutaneous neuromodulation in treating constipation and retentive fecal incontinence in children and adolescents. Methods: A systematic review of randomized clinical trials was performed. Medline (PubMed), PEDro, SciELO, Cochrane (CENTRAL), Embase, and Scopus databases were searched from March 2000 to August 2022. We included clinical trials evaluating transcutaneous neuromodulation in children with constipation and fecal incontinence compared or associated with other types of treatment. Two reviewers independently selected relevant studies, assessed the methodological quality, and extracted the data. Results: Three studies with 164 participants were included in this review. Two meta-analyses were generated based on these studies. These analyses revealed that transcutaneous neuromodulation is an effective adjuvant treatment modality that improves children’s constipation and retentive fecal incontinence. The methodological quality of the included studies was classified as high based on the assessment of the quality of evidence, with a high degree of confidence based on the GRADE system. Conclusions: Transcutaneous neuromodulation is an effective adjuvant treatment modality for children with constipation and retentive fecal incontinence.

## 1. Introduction

Constipation is a multifactorial disorder with varied clinical presentations in children, including infrequent evacuation, straining, the elimination of bulky feces, and retentive fecal incontinence. In addition, these symptoms correlate with psychosocial disorders that significantly affect the quality of life [1]. In children, most cases are functional, with no anatomical or biochemical causes, and are mainly associated with evacuation dynamics and dietary patterns. Genetic, psychological, and behavioral factors and characteristics related to the intestinal microbiome, colonic motility, and anorectal function may also be responsible for constipation. The stool accumulation in the rectum causes difficulties in defecation, abdominal distention, pain, and retentive fecal incontinence due to the overflow of feces [1,2,3].

The treatment of constipation is divided into four steps: (1) education, (2) fecal disimpaction, (3) reconditioning normal bowel habits, and (4) preventing recurrence [2,3]. Children with constipation typically respond well to conventional treatment. However, patients may have unsatisfactory responses or report only minor improvements in their symptoms. Cases with these responses are considered to be refractory to treatment. Unfortunately, the treatment options for refractory cases are few, with varying results. Therefore, recent guidelines recommend alternatives, such as botulinum toxin injection into the anal sphincter, retrograde or anterograde intestinal lavages, acupuncture, fecal microbiota transplantation, ostomies, colorectal resections, and neuromodulation [2,3].

Transcutaneous neuromodulation is used in pediatrics to treat several diseases. In addition, transcutaneous neuromodulation effectively treats cases, including constipation and retentive fecal incontinence, which are refractory to conventional medicine [4]. In 2016, a systematic review evaluated the effectiveness and safety of transcutaneous neuromodulation in treating constipation in children [5]. Unfortunately, the review failed to identify the effectiveness and safety of transcutaneous neuromodulation due to insufficient evidence. Consequently, the authors suggested using randomized controlled trials to evaluate the effectiveness of transcutaneous neuromodulation in managing constipation in children [5]. A recent systematic review assessed a variety of methodological designs and scenarios to analyze the effects of electrical stimulation in children diagnosed with constipation and fecal incontinence [6]. Although this study showed positive results, meta-analyses were not performed [6]. Thus, the efficacy and potential benefits of neuromodulation therapy in children with constipation have yet to be elucidated.

Therefore, this review aimed to perform a systematic review and meta-analysis of the literature to assess the effectiveness of varying transcutaneous neuromodulation modalities to improve constipation and retentive fecal incontinence (encopresis) in children and adolescents with functional constipation and compare transcutaneous neuromodulation modalities with other interventions or sham procedures.

## 2. Patients and Methods

A systematic review and meta-analysis of available randomized clinical trials were performed on children and adolescents with functional constipation or retentive fecal incontinence subjected to transcutaneous neuromodulation. The review and meta-analysis followed the Preferred Reporting Items for Systematic Reviews and Meta-Analyses statement (PRISMA) [7] and the Cochrane recommendations for systematic reviews [8]. The study was designed according to the PICO acronym: P (population): patients up to 18 years old with functional constipation, with or without retentive fecal incontinence; I (intervention): transcutaneous neuromodulation; C (comparison): between transcutaneous neuromodulation and other interventions or sham procedures; O (outcome): effectiveness in improving constipation and retentive fecal incontinence (encopresis).

### 2.1. Eligibility Criteria

This review included randomized clinical trials with children and adolescents (up to 18 years old) diagnosed with functional constipation based on the Rome III or IV criteria [9,10,11], with or without retentive fecal incontinence, subjected to any technique of transcutaneous neuromodulation, associated or not with other types of treatment. The studies also had to include comparisons to other interventions or sham procedures, which reported effectiveness in improving constipation and retentive fecal incontinence. Studies with other methodological designs were excluded, including systematic, bibliographic, or integrative reviews, case studies, cohort studies, studies with animals, in vitro studies, and expert opinion studies.

### 2.2. Data Sources and Searches

The search period started in March 2000 and ended in August 2022. Only published studies written in English, Spanish, or Portuguese were included. Studies were obtained from the Medical Literature Analysis and Retrieval System Online (MEDLINE^®^/PubMed^®^), Physiotherapy Evidence Database (PEDro), Scientific Electronic Library Online (SciELO), Embase, Scopus, and the Cochrane Library. A detailed search of titles and abstracts was performed using the following keywords: “constipation” OR “colonic inertia” OR “dyschezia” AND “fecal incontinence” OR “bowel incontinence” OR “fecal soiling” AND “electrical stimulation” OR “electrical stimulation therapy” OR “transcutaneous electrical stimulation”. Duplicate publications were excluded, and electronic databases were last accessed on 31 August 2022.

### 2.3. Study Selection and Data Extraction

Two authors (R.R.C and L.F.O.G.) independently reviewed the extracted studies by title, keywords, and abstract. Disagreements were resolved by a third (P.L.T.A.L) and a fourth (E.V.P.O) reviewers. Full-text reviews were performed on all of the included articles. The studies were independently reviewed by two authors (R.R.C and L.F.O.G.) for data collection using a standardized form (Supplementary Material File S1) and the risk of bias assessment.

### 2.4. Statistical Analysis and Risk of Bias

All analyses were performed using Review Manager (RevMan) [computer program]. Version 5.4, The Cochrane Collaboration, 2020.

Dichotomous outcome data were used to calculate the hazard ratio and corresponding 95% confidence interval. Forest plot charts summarized the data. Inconsistencies between studies summed up in meta-analyses were quantified using heterogeneity tests. I^2^ statistic tests were used to determine heterogeneity. Statistical significance was assumed when I^2^ was >50%, with a value of *p* < 0.1. Funnel plots were used to determine potential publication bias [12].

The quality of risk bias of the included studies was assessed using the Cochrane Handbook criteria. The criteria for analysis included: selection bias, random sequence generation, allocation concealment, performance bias, blinding of participants and personnel, detection bias, blinding of outcome assessors, attrition bias, incomplete outcome data, reporting bias, selective outcome reporting, other biases, and other sources of bias [12].

### 2.5. Quality of Evidence and Level of Recommendation

The principles of the Grading of Recommendations Assessment Development Evaluation (GRADE) system were used to interpret the quality of evidence and recommendation level [13].

### 2.6. Declaration and Registration

This systematic review and meta-analysis was registered on 28 April 2020 on the International Prospective Register of Systematic Reviews platform, using the following registration number CRD42020153176. Financial support was not provided for this study.

## 3. Results

### 3.1. Study Selection

**Figure 1:**
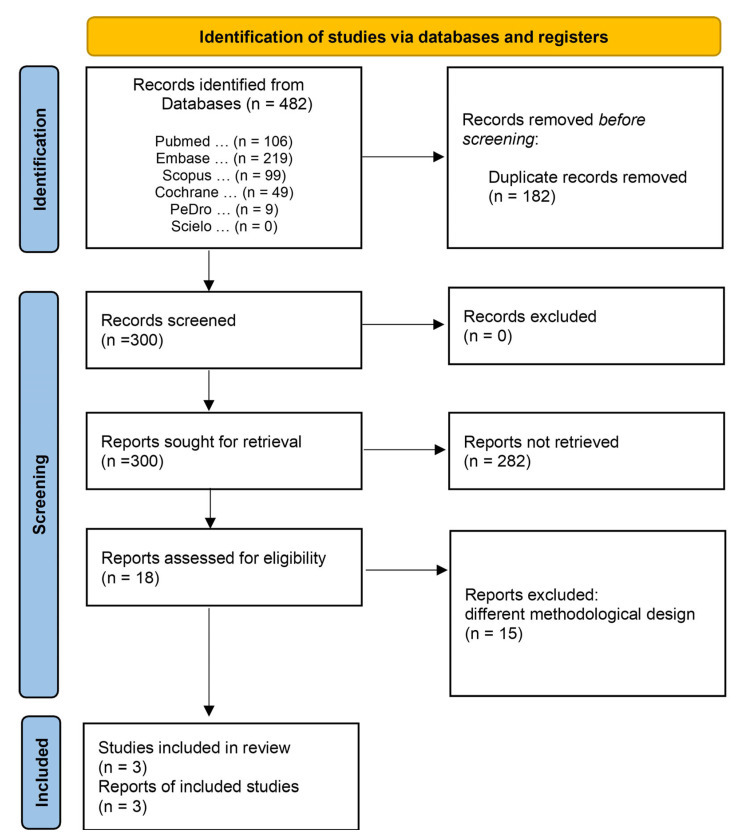
Access and selection flow diagram of the inclusion and exclusion studies.

A total of 482 titles, 106 of which were published in PubMed, 219 studies in Embase, 99 in Scopus, 49 in Cochrane, 9 in PEDro, and none in SciELO, were identified in the search. After removing 182 duplicates, 300 studies met the minimum criteria, and were considered potential references. A second analysis revealed that 282 studies were excluded for failing to meet the inclusion criteria. Eighteen studies met the thematic criteria and underwent qualitative analysis. After a detailed analysis, 15 studies were excluded: 7 non-randomized studies, 2 pilot studies, 3 literature reviews, 1 prospective cohort study, 1 retrospective study, and 1 randomized clinical trial (excluded due to the absence of results for the outcomes of interest) (Figure 1). The characteristics of the excluded studies are presented in Supplementary Material File S2. Finally, three studies were included in the meta-analyses: de Abreu et al., 2021 [14], Seyedian et al., 2020 [15] and Sharifi-Rad et al., 2018 [16].

### 3.2. Characteristics of the Included Studies

A total of 164 children were included in the study; 90 from the study by Sharifi-Rad et al., 2018 [16], 34 from Seyedian et al., 2020 study [15], and 40 from that by de Abreu et al., 2021 [14]. Intervention options included studies by Seyedian et al., 2020 [15] and Sharifi-Rad et al., 2018 [16] in which transcutaneous interferential neuromodulation associated with pelvic floor muscle (PFM) exercises was compared to sham transcutaneous neuromodulation associated with PFM exercises or PFM exercises alone. Both studies used transcutaneous interferential electrical stimulation with self-adhesive electrodes placed on the pelvic [15] or abdominal skin [16]. Similarities were also noted in the pulse duration, frequency, and scan coverage parameters and PFM exercises. PFM exercises included a regular exercise program of at least 15 min/day with muscle contraction for 10 s, followed by relaxation for 30 s. De Abreu et al., 2021 [14] compared transcutaneous parasacral neuromodulation associated with standard urotherapy to sham transcutaneous neuromodulation associated with standard urotherapy. Parasacral transcutaneous electrical nerve stimulation was performed with self-adhesive electrodes placed on parasacral skin. Standard urotherapy consisted of urinary behavioral guidelines and dietary guidelines.

The studies by Abreu et al., 2021 [14], Seyedian et al., 2020 [15], and Sharifi-Rad et al., 2018 [16] evaluated the following primary outcomes: (1) the number of patients with or without constipation according to the Rome III or IV criteria and (2) the number of patients with or without fecal incontinence (encopresis).

### 3.3. Meta-Analysis on Constipation Improvement

**Figure 2:**
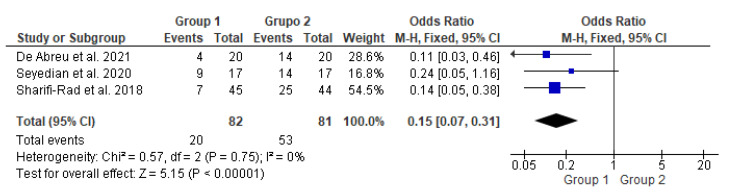
Meta-analysis comparing constipation improvement in patients who underwent transcutaneous neuromodulation associated with other therapies (Group 1) and those who underwent other therapies with or without sham (Group 2) [14,15,16].

This meta-analysis (Figure 2) included three studies [14,15,16] with a total of 163 participants. It compared two groups: patients who underwent transcutaneous neuromodulation associated with other therapies (Group 1) and those who underwent other therapies with or without transcutaneous neuromodulation sham (Group 2). Group 1 included patients who underwent neuromodulation associated with standard urotherapy [14] or PFM exercises [15,16], whereas Group 2 included those who underwent standard urotherapy [11] or PFM exercises [15,16] without neuromodulation [15] or with sham neuromodulation [14,16].

In Figure 2, the study by Seyedian et al., 2020 [15] touched on the null hypothesis line. However, studies by de Abreu et al., 2021 [14], Sharifi-Rad et al., 2018 [16], and the diamond demonstrated the effectiveness of transcutaneous neuromodulation as an adjuvant method (Group 1) in improving constipation compared to other therapies with or without sham (Group 2). Regarding the number of events over the total number of participants (dichotomous statistical outcome), Group 2 had the highest number of patients with constipation.

### 3.4. Meta-Analysis on Fecal Incontinence Improvement

**Figure 3:**
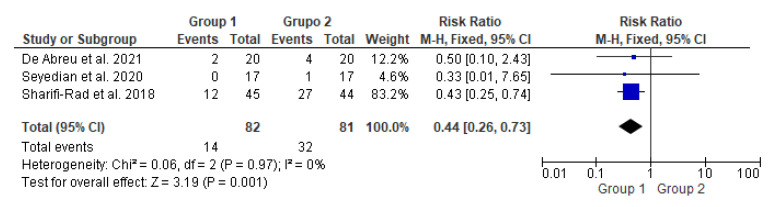
Meta-analysis comparing fecal incontinence improvement in patients who underwent transcutaneous neuromodulation associated with other therapies (Group 1) and those who underwent other therapies with or without sham (Group 2) [14,15,16].

This meta-analysis (Figure 3) included three studies with a total of 163 participants. It compared two groups: patients who underwent transcutaneous neuromodulation associated with other therapies (Group 1) and those who underwent other therapies with or without transcutaneous neuromodulation sham (Group 2). Group 1 included patients who underwent neuromodulation associated with standard urotherapy [14] or PFM exercises [15,16], whereas standard urotherapy [14] or PFM exercises [15,16] without neuromodulation [15] or with sham neuromodulation [14,16].

In Figure 3, the studies by de Abreu et al., 2021 [14] and Seyedian et al., 2020 [15] touched on the null hypothesis line. However, studies by Sharifi-Rad et al., 2018 [16] and the diamond demonstrated the effectiveness of transcutaneous neuromodulation as an adjuvant method (Group 1) in improving fecal incontinence compared to other therapies with or without sham (Group 2). Regarding the number of events over the total number of participants (dichotomous statistical outcome), Group 2 had the highest number of patients with fecal incontinence.

### 3.5. Publication Bias Analysis Using Funnel Plots

**Figure 4:**
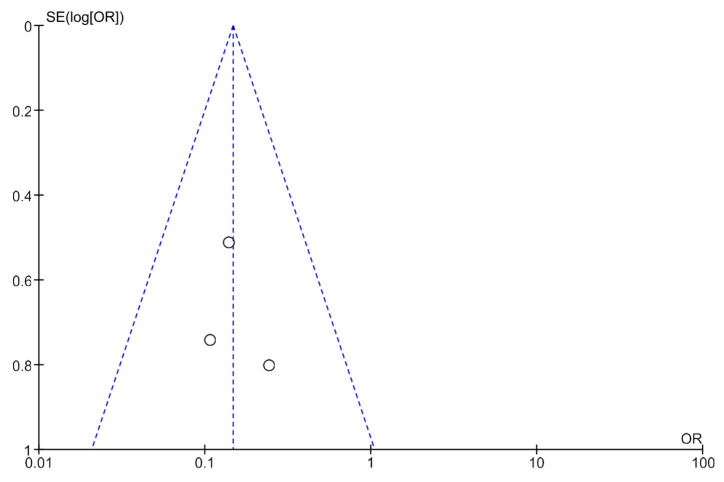
A funnel plot showing the meta-analysis of transcutaneous neuromodulation associated with other therapies versus other therapies with or without sham for the constipation outcome.

**Figure 5:**
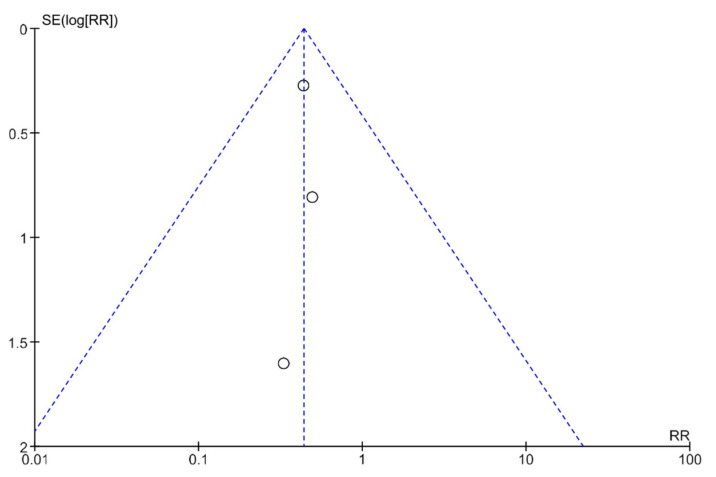
A funnel plot showing the meta-analysis of transcutaneous neuromodulation associated with other therapies versus other therapies with or without sham for the fecal incontinence outcome.

Publication bias was analyzed using funnel plots (Figure 4 and Figure 5). The symmetry of the plots suggests the absence of publication bias.

### 3.6. Risk of Bias and Methodological Quality Analysis

**Figure 6:**
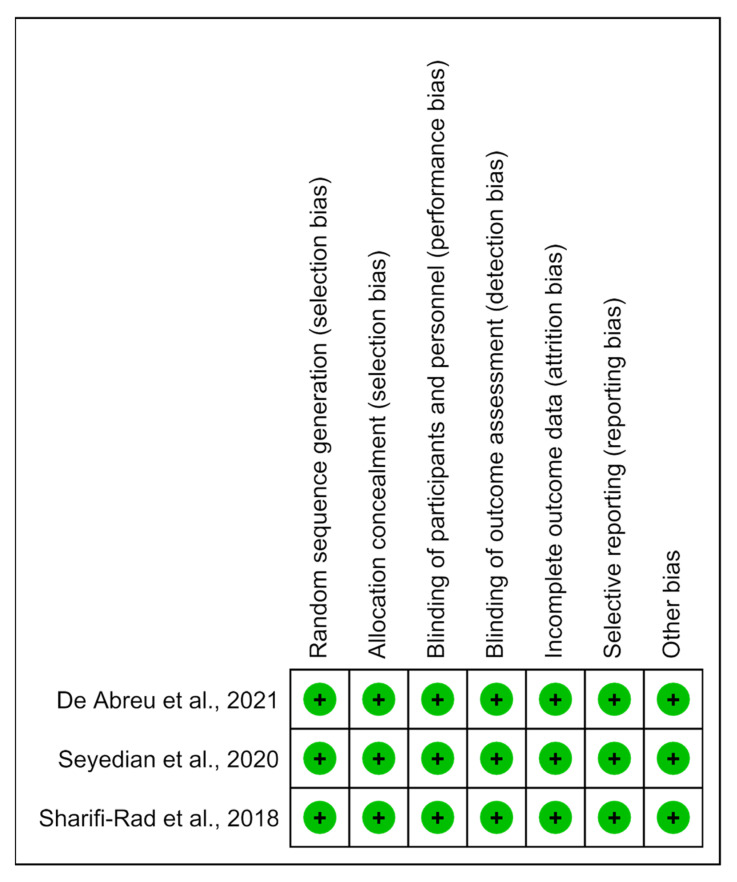
Risk of bias summary: analysis of the authors’ judgments on each risk of bias item for the included studies [14,15,16].

The methodological quality of the studies was predominantly determined to have a low risk of bias (Figure 6).

### 3.7. Quality of Evidence and Level of Recommendation

**Figure 7:**
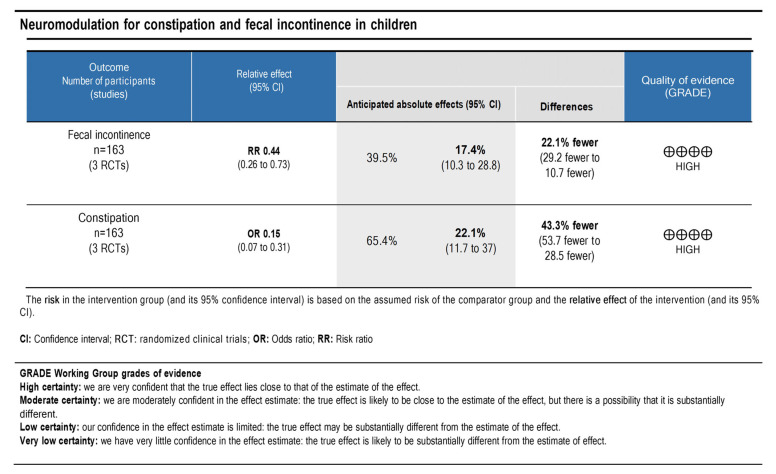
The quality of scientific evidence and the strength of recommendation for fecal incontinence and constipation outcomes.

The principles of the GRADE system were used to analyze the quality and body of evidence associated with the specific outcomes (the number of patients with constipation and with fecal incontinence that improved). A GRADE table [13] (Figure 7) was constructed to summarize the findings of the analysis. The fecal incontinence and constipation showed high certainties regarding the level of evidence. Therefore, a high degree of confidence was demonstrated with the true effect being close to the estimate.

## 4. Discussion

In recent decades, studies have focused on neurostimulation as a new strategy to treat a variety of symptoms including nausea, vomiting, and intestinal and urinary disorders. In addition, using this modality as adjuvant therapy for children with gastrointestinal disorders, including constipation refractory to conventional treatment, has increased [4,6].

Transcutaneous neuromodulation is a less invasive technique with fewer complications, and decreased costs compared with other neuromodulation modalities [4,6]. Thus, the present review specifically analyzed the use of transcutaneous neuromodulation techniques to treat constipation in children and adolescents. Transcutaneous neuromodulation was proven to be an effective adjuvant therapy that improved constipation and retentive fecal incontinence in children and adolescents. This effect was identified through a comparative analysis between transcutaneous neuromodulation associated with other forms of treatment (PFM exercises and standard urotherapy) and control or sham control groups.

Transcutaneous neuromodulation was achieved by alternating currents (interferential) through expensive and complex devices; or by pulsing a current through simpler, portable, cheaper, and readily available devices. The simpler devices can be used at home by the parents or guardians following specialist training [4,5,6]. In this systematic review, two of the three included studies used transcutaneous neuromodulation with interferential current [15,16], whilst pulsed current with sacral stimulation was used in the third study [14]. These studies were compared based on a joint analysis of their respective transcutaneous neuromodulation modalities. Transcutaneous neuromodulation acts at different neural levels to restore the balance between excitatory and inhibitory regulations in the central and peripheral nervous systems [17]. A current is used to stimulate nerve fibers, which results in parasympathetic activation through deep stimulation, and improves peristalsis. Studies have shown that the stimulation pathway is modulated by the vagus nerve [18]. Activation is initiated by the stimulation of sensory and non-motor fibers, which increases bladder and rectal filling perception [19]. The cingulate gyrus, sensorimotor cortex, and mesencephalon act on the progressive sensation of fullness and modulate efferent impulses and voiding and defecation reflexes [15,16,20].

Constipation typically presents with urinary symptoms in children, characterizing the “Bladder and Bowel Dysfunction” (BBD) spectrum. BBD is a common and possibly underdiagnosed entity in children and consists of lower urinary tract symptoms, such as urinary incontinence, urgency, hesitancy, and dysuria, associated with intestinal complaints, including constipation or retentive fecal incontinence [21]. The pathophysiological basis for BBD consists of embryological, anatomical, and functional interactions between the bladder and the intestine. Feces accumulation in the rectum affect the emptying and retention capacity of the bladder, either by mechanical compression or by neural stimuli changes in the bladder and pelvic floor muscles. In contrast, voluntary urinary retention leads to a reduced sensation of bowel movement, resulting in constipation or encopresis [22]. These patients require a complex and multidisciplinary treatment that involves behavioral measures (standard urotherapy), drugs, and adjuvant therapies such as neuromodulation and biofeedback [21]. Two of the three studies included in this review [14,15] analyzed patients with lower urinary tract symptoms and BBD whose diagnosis of constipation was established based on the Rome IV criteria [9,10].

Pelvic floor muscle exercises are simple exercises that increase the child’s awareness of pelvic musculature and the synergistic abdominal and perineal action. These exercises teach the child to relax these muscles during defecation [23]. Subsequent to training with a physical therapist, patients repeat these exercises at home. Therapy reduced constipation symptoms in children with constipation or BBD and is, therefore, an effective adjuvant treatment option for children and adolescents with constipation [16,24]. Similarly, two of the reviewed studies had also used PFM exercises to alleviate constipation [15,16].

The primary limitation of our review is related to the paucity of randomized clinical trials evaluating the effects of transcutaneous neuromodulation in children with constipation and fecal incontinence. For this reason, it was impossible to assess the impact of transcutaneous neuromodulation alone in this setting. In contrast, one of the strong points of the present systematic review is the method used, which is consistent, judicious, and follows the main methodological guidelines [8]. The literature search was comprehensive, used various databases, and identified relevant studies with good methodological quality. In addition, two meta-analyses on the primary clinical outcomes of constipation and retentive fecal incontinence were performed in this review; meta-analysis had not been performed in previously published studies on this topic. Publication bias was not noted in the included studies based on the results of the funnel plots. The heterogeneity of the meta-analysis was considered null, thereby strengthening the evidence and recommendation levels of the results. The methodological quality of the included studies was classified as high based on the evidence quality assessment. The method was determined to have a high confidence using the GRADE system. These results verified the effectiveness of transcutaneous neuromodulation for the first time as adjuvant therapy to treat children with constipation.

## 5. Conclusions

Transcutaneous neuromodulation showed effectiveness as an adjuvant treatment modality for children with constipation. The association of transcutaneous neuromodulation with other treatment modalities (PFM exercises or standard urotherapy) showed significant efficacy in improving constipation and retentive fecal incontinence. Evaluations of these primary outcomes verified the results. Therefore, this review has a high degree of scientific evidence and strength of recommendation for using neuromodulation as an adjuvant method to treat constipation and retentive fecal incontinence in children and adolescents.

### Implications for Clinical Practice

The use of transcutaneous neuromodulation as an adjuvant method to treat constipation and retentive fecal incontinence in children is highly recommended.

## Data Availability

The data presented in this study are available on request from the corresponding author. The data are not publicly available due to privacy.

## Supplementary Materials

The following supporting information can be downloaded at: https://www.mdpi.com/article/10.3390/life13020430/s1, File S1: Data extraction form; File S2: Characteristics of excluded studies. References [25,26,27,28,29,30,31,32,33,34,35,36] has cited in Supplementary Materials.
