# Supplementary material for: Transcutaneous Neuromodulation for Constipation and Fecal Incontinence in Children: A Systematic Review and Meta-Analysis
Source: Life (Basel). 2023 Feb 2;13(2):430. doi: 10.3390/life13020430 (PMC9960109; doi:10.3390/life13020430)
Supplement: Supplementary file 1 [file life-13-00430-s001.zip › Supplementary material file S1.pdf]

## DATA EXTRACTION FORM | COCHRANE HADBOOK

*ID – Author, year of publication:* ***Sharifi-Rad et al., 2018***

What the author will be asked;

**METHOD**

1. Design: **Randomized clinical trial.**

2. Multicentric or single-centre: **Single-centre – in Tehran, Iran.**

3. Study period: **January 2014 to April 2017, corresponding to 40 months.**

4. Justification for the size of the sample: **no report.**

5. Allocation generation: **Computer random number generation. (Low risk of bias).**

6. Results of allocation: **envelope numbered sequences, opaque and sealed. (Low risk of bias).**

7. Blinding of Participants and Investigators: **Blinding of participants and practitioners secured and blinding is unlikely to have been blinded. (Low risk of bias).**

8. Outcome rater blinding: **Blinding of outcome raters and was performed, and it is unlikely that the blinding was broken. (Low risk of bias).**

9. Incomplete outcome data: **there was no loss of outcome data. (Low risk of bias).**

10. Selective reporting: **the study protocol is available and all pre-specific outcomes that are of interest were reported as proposed. (Low risk of bias). The study appears to be free from other sources of bias. (Low risk of bias).**

11. Other biases: **the study appears to be free from other sources of bias. (Low risk of bias).**

**PARTICIPANTS**

1. N: **90 participants**.

2. Gender: **46 boys and 44 girls**.

3. Age (average): **6.5 + 2.3 years.**

4. Study scenario: **Neuromodulation (interferential current) versus pelvic floor muscle training associated with neuromodulation (interferential current).**

5. Inclusion criteria: **Pediatric patients who had constipation, defecation frequency less than three times a week, positive history of passing hard stools, episodes of fecal soiling, abnormal stool shape, and painful defecation.**

6. Exclusion criteria: **inflammatory and metabolic diseases, neurological and psychiatric disorders, Hirschsprung's disease and also a positive history of abdominal or anal sphincter surgery.**

**INTERVENTION**

1. Experimental group: **Neuromodulation (interferential current).**

7. Control group: **pelvic floor muscle training associated with neuromodulation (interferential current).**

EVALUATED OUTCOMES

1. Outcome: **The primary outcome was defined as the absence of functional constipation according to the Rome III criteria. Secondary outcomes were measuring an increase in defecation frequency of twice a week more than baseline, absence of episodes of fecal soiling, absence of abnormal stool shape, measurement of pain, and constipation scores. Changes in constipation-related QoL scores were also compared between the two groups**.

**NOTE**

1. Observations: **does not report.**

**DATA EXTRACTION FORM | COCHRANE HADBOOK**

***ID – Author, year of publication: de Abreu et al., 2021***

What the author will be asked;

**METHOD**

1. Design: **randomized clinical trial.**

2. Multicentric or single-centre: **Single-centre - Center for Urinary Disorders of Children (CEDIMI), Bahia School of Medicine and Public Health, Salvador, Bahia, Brazil.**

3. Study period: **May 2015 to February 2018, totaling 26 months.**

4. Justification for the size of the sample: **no report.**

5. Allocation generation: **Computer random number generation. (Low risk of bias).**

6. Results of allocation: **envelope numbered sequences, opaque and sealed. (Low risk of bias).**

7. Blinding of Participants and Investigators: **Blinding of participants and practitioners secured and blinding is unlikely to have been blinded. (Low risk of bias).**

8. Outcome rater blinding: **Blinding of outcome raters and was performed, and it is unlikely that the blinding was broken. (Low risk of bias).**

9. Incomplete outcome data: **there was no loss of outcome data. (Low risk of bias).**

10. Selective reporting: **the study protocol is available and all pre-specific outcomes that are of interest were reported as proposed. (Low risk of bias). The study appears to be free from other sources of bias. (Low risk of bias).**

11. Other biases: **the study appears to be free from other sources of bias. (Low risk of bias).**

**PARTICIPANTS**

1. N: **40 participants.**

2. Gender: **does not report.**

3. Age (average): **5 – 7 years.**

4. Study scenario: **Neuromodulation (PTNS) versus Sham Group.**

5. Inclusion criteria: **patients between 5 and 17 years of age seeking urology treatment with bladder and bowel dysfunction, defined as the presence of functional constipation associated with lower urinary tract symptoms, without previous treatment.**

6. Exclusion criteria: **patients with neurological and/or anatomical alterations of the urinary and/or digestive tract, unable to attend treatment sessions 3 times a week, with diabetes mellitus and/or diabetes insipidus and using anticholinergics or laxatives .**

**INTERVENTION**

1. Experimental group: **Neuromodulation (PTNS).**

2. Control group: **Sham group.**

**EVALUATED OUTCOMES**

1. Outcome: **Rome IV criteria assess functional bowel constipation (1. frequency of bowel movements; 2. difficulty or pain when defecating; 3. sensation of incomplete evacuation; 4. abdominal pain; 5. time spent on the toilet >5 minutes; 6. use of laxatives or digital assistance; 7. Failed evacuation attempts for 24 hours; and 8. Constipation (symptom duration). The Bristol Stool Scale. Ultrasonography evaluated post-void residual urine and rectal diameter.**

NOTE

1. Observations: **does not report**

**DATA EXTRACTION FORM | COCHRANE HADBOOK**

***ID – Author, year of publication: Ladi-Seyedian et al., 2020***

What the author will be asked;

**METHOD**

1. Design: **Randomized clinical trial.**

2. Multicentric or single-centre: **Single-centre – in Tehran, Iran.**

3. Study period: **May 2015 to February 2018, corresponding to 26 months.**

4. Justification for the size of the sample: **no report.**

5. Allocation generation: **Computer random number generation. (Low risk of bias).**

6. Results of allocation: **envelope numbered sequences, opaque and sealed. (Low risk of bias).**

7. Blinding of Participants and Investigators: **Blinding of participants and practitioners secured and blinding is unlikely to have been blinded. (Low risk of bias).**

8. Outcome rater blinding: **Blinding of outcome raters and was performed, and it is unlikely that the blinding was broken. (Low risk of bias).**

9. Incomplete outcome data: **reasons for loss of data may be related to the outcome investigated, with imbalance in the number of patients or reasons for loss between intervention groups. (losses less than 20%). (low risk of bias).**

10. Selective reporting: **the study protocol is available and all pre-specific outcomes that are of interest were reported as proposed. (Low risk of bias). The study appears to be free from other sources of bias. (Low risk of bias).**

11. Other biases: **the study appears to be free from other sources of bias. (Low risk of bias).**

PARTICIPANTS

1. N: **34 participants.**

2. Gender: **6 boys and 28 girls.**

3. Age (average): **7.4 + 2.2 years.**

4. Study scenario: **Neuromodulation (interferential current) versus pelvic floor muscle training associated with neuromodulation (interferential current).**

5. Inclusion criteria: **children older than 5 years, history of lower urinary tract dysfunction based on clinical symptoms, fulfillment of diagnostic criteria for constipation according to Rome IV criteria.**

6. Exclusion criteria: **neuropathic disease, anatomical defects and mental retardation.**

**INTERVENTION**

1. Experimental group: **Neuromodulation (interferential current).**

1. Control group: **pelvic floor muscle training associated with neuromodulation (interferential current).**

**EVALUATED OUTCOMES**

1. Outcome: **using subjective parameters (presence or absence of constipation, daytime incontinence, nighttime wetting and number of wetting episodes).**

**NOTE**

1. Observations: **does not report.**
