# Supplementary material for: Transcutaneous Neuromodulation for Constipation and Fecal Incontinence in Children: A Systematic Review and Meta-Analysis
Source: Life (Basel). 2023 Feb 2;13(2):430. doi: 10.3390/life13020430 (PMC9960109; doi:10.3390/life13020430)
Supplement: Supplementary file 1 [file life-13-00430-s001.zip › Supplementary material file S2.pdf]

Characteristics of excluded studies [ordered by study ID]

Study	Reason for exclusion
Chase 2005 [2]	This was a single-group pilot study of eight children (7-16 years) assessing the effects of TESIC in children who had chronic treatment-resistant constipation and soiling who had failed various treatment options<br><br>Excluded on the basis of study design
Chase 2009 [3]	This was a non-randomised pilot study that assessed the effectiveness of daily home TESIC in improving the bowel habits of children with slow-transit constipation<br><br>This study recruited children who were poor responders in a previous RCT on TESIC and slow-transit constipation<br><br>Excluded on the basis of study design
Clarke 2012 [4]	This was a non-randomised single-group before-and-after study evaluating the colonic propagating sequence of a group of children with slow-transit-constipation following the application of TESIC<br><br>Excluded on the basis of study design
Dwyer 2014 [5]	A prospective cohort study that evaluated the use of sacral neuro-modulation in children with dysfunctional elimination syndrome, which included bowel or bladder dysfunction<br><br>Excluded on the basis on study design and population assessed
Ismail 2009 [3]	A non-randomised follow-up study that evaluated the effectiveness of daily TES application at home in participants who responded poorly to thrice-weekly application of TES in an RCT<br><br>Excluded on the basis of study design
Moeller 2015 [1]	A randomised double-blind controlled trial that evaluated the acute effect of transcutaneous electrical nerve stimulation on rectal motility in children with overactive bladder<br><br>Excluded on the basis of population
Thomas 2013 [6]	A literature review assessing the effect of sacral nerve stimulation in adults and children with slow-transit constipation<br><br>Excluded on the basis of article type
Iacona 2019 [12]	A systematic review of the literature on the use of neuromodulation in children for constipation and soiling refractory to medical treatment.<br>Excluded based on article type

Study	Reason for exclusion
R. T. Ng 2016 [13]	A systematic review of the literature evaluating the efficacy and safety of TENS (Transcutaneous electrical nerve stimulation) when used to improve bowel function and constipation-related symptoms in children with constipation.<br>Excluded based on article type
Yik 2012a [7]	This was a non-randomised single-group before-and-after study that evaluated the colonic propagating sequence of a group of children with slow-transit-constipation following the application of TESIC<br><br>Excluded on the basis of study design
Yik 2012b [8]	This was a non-randomised single-group before-and-after study that evaluated the clinical symptoms of a group of children with slow-transit-constipation following the application of daily TES at home in addition to laxative treatment<br><br>Excluded on the basis of study design
Yik 2012c [9]	This retrospective audit aimed to determine if TES use affected appendicostomy formation rates<br><br>Excluded on the basis of study design
Yik 2013a [10]	A single-group before-and-after study that assessed the effects of TES delivered initially in the physical therapist's clinic for 4 weeks then at home in 62 children with clinical symptoms related to constipation<br><br>Published in abstract form<br><br>Excluded on the basis of study design
Yik 2013b [11]	A single-group non-comparative study that assessed the effects of TES initially at physical therapist's clinic for 4 weeks then at home for 3-6 months in 62 children with clinical symptoms related to constipation<br><br>Published in abstract form<br><br>The data set reported were similar to (Yik 2013a)<br><br>Excluded on the basis of study design

TESIC: transcutaneous electrical stimulation using interferential current.

TES: transcutaneous electrical stimulation.

References to studies excluded from this review

[1] I. Moeller Joensson, S. Hagstroem, C. Siggaard, W. Bower, J. C. Djurhuus, e K. Krogh, “Transcutaneous Electrical Nerve Stimulation Increases Rectal Activity in Children”, *J Pediatr Gastroenterol Nutr*, vol. 61, nº 1, p. 80–84, 2015, doi: 10.1097/MPG.0000000000000802.

[2] J. Chase, V. J. Robertson, B. Southwell, J. Hutson, e S. Gibb, “Pilot study using transcutaneous electrical stimulation (interferential current) to treat chronic treatment-resistant constipation and soiling in children”, *J. Gastroenterol. Hepatol.*, vol. 20, nº 7, p. 1054–1061, 2005, doi: 10.1111/J.1440-1746.2005.03863.X.

[3] K. A. Ismail *et al.*, “Daily transabdominal electrical stimulation at home increased defecation in children with slow-transit constipation: a pilot study”, *J. Pediatr. Surg.*, vol. 44, nº 12, p. 2388–2392, dez. 2009, doi: 10.1016/J.JPEDSURG.2009.07.063.

[4] M. C. Clarke *et al.*, “Transabdominal electrical stimulation increases colonic propagating pressure waves in paediatric slow transit constipation”, *J Pediatr Surg*, vol. 47, nº 12, p. 2279–2284, 2012, doi: 10.1016/j.jpedsurg.2012.09.021.

[5] M. E. Dwyer, D. R. Vandersteen, P. Hollatz, e Y. E. Reinberg, “Sacral neuromodulation for the dysfunctional elimination syndrome: a 10-year single-center experience with 105 consecutive children”, *Urology*, vol. 84, nº 4, p. 911–918, out. 2014, doi: 10.1016/J.UROLOGY.2014.03.059.

[6] G. P. Thomas, T. C. Dudding, G. Rahbour, R. J. Nicholls, e C. J. Vaizey, “Sacral nerve stimulation for constipation”, 2012, doi: 10.1002/bjs.8944.

[7] Y. I. Yik, K. A. Ismail, J. M. Hutson, e B. R. Southwell, “Home transcutaneous electrical stimulation to treat children with slow-transit constipation”, *J Pediatr Surg*, vol. 47, nº 6, p. 1285–1290, 2012, doi: 10.1016/j.jpedsurg.2012.03.037.

[8] Y. I. Yik, L. C. Y. Leong, J. M. Hutson, e B. R. Southwell, “The impact of transcutaneous electrical stimulation therapy on appendicostomy operation rates for children with chronic constipation--a single-institution experience”, *J. Pediatr. Surg.*, vol. 47, nº 7, p. 1421–1426, jul. 2012, doi: 10.1016/J.JPEDSURG.2012.01.017.

[9] Y. I. Yik, K. A. Ismail, J. M. Hutson, e B. R. Southwell, “Tu1366 Daily Transabdominal Electrical Stimulation Reduces Soiling in Children With Slow-Transit Constipation”, *Gastroenterology*, vol. 142, nº 5, p. S-812, maio 2012, doi: 10.1016/S0016-

5085(12)63160-6.

[10] Yik YI, Ismail K, Hutson J, Southwell B. Home-based transabdominal electrical stimulation slowly improves treatment-resistant chronic constipation in children. Neuromodulation. 2013;16:e102.

[11] Yik YI, Ismail KA, Hutson JM, Southwell BR. Transabdominal electrical stimulation using interferential current is able to treat chronic constipation in children. Neuromodulation. 2013;16:e13.

[12] R. Iacona, L. Ramage, e G. Malakounides, "Current State of Neuromodulation for Constipation and Fecal Incontinence in Children: A Systematic Review", *Eur J Pediatr Surg*, vol. 29, nº 6, p. 495–503, 2019, doi: 10.1055/s-0038-1677485.

[13] R. T. Ng, W. S. Lee, H. L. Ang, K. M. Teo, Y. I. Yik, e N. M. Lai, "Transcutaneous electrical stimulation (TES) for treatment of constipation in children", *Cochrane Database Syst. Rev.*, 2016, doi: 10.1002/14651858.cd010873.pub4

[14] Southwell B. Transabdominal electrical stimulation to treat slow transit constipation in children. Neuromodulation 2012;15:63.
